# Malathion biodegradation and biotransformation in water and sediment systems: bacterial pathways, environmental drivers, ecological impacts, and bioremediation strategies

**DOI:** 10.1007/s11274-026-05239-8

**Published:** 2026-09-06

**Authors:** Gayatri Basapuram, Srimanti Duttagupta, Avishek Dutta

**Affiliations:** 1 Department of Geology, University of Georgia, Athens, GA 30602 USA; 2 Savannah River Ecology Laboratory, University of Georgia, Aiken, SC 29802 USA

**Keywords:** Biotransformation, Malathion, Sediment, Water, Environmental parameters, Bioremediation

## Abstract

Malathion is one of the most widely used organophosphate insecticides, and its environmental fate is strongly influenced by microbial transformation in the water column and sediments. This review synthesizes current knowledge of the enzymatic and metabolic pathways that govern the persistence of malathion in aquatic, sedimentary, and wastewater systems. Members of the genera *Pseudomonas*, *Bacillus*, *Acinetobacter*, *Rhodococcus*, *Micrococcus*, and several other commonly studied taxa produce enzymes such as carboxylesterases and hydrolases that cleave the carboxyl ester linkage. The major metabolites formed are malathion monocarboxylic acid (MMC) and malathion dicarboxylic acid (MDC), which are generally far less toxic than the parent compound and, under favorable conditions, can be further mineralized to carbon dioxide and inorganic phosphate. A parallel transformation route, oxidative desulfuration, can generate malaoxon, a metabolite substantially more toxic and persistent than malathion and therefore important to consider in environmental risk assessment. Degradation rates are typically highest at 25–35 °C and neutral to slightly alkaline pH conditions that favor hydrolytic activity. Redox conditions further influence pathway predominance: oxic environments generally favor hydrolysis and oxidative transformation, whereas anoxic environments may slow overall degradation and promote alternative reductive processes. Nutrient availability can stimulate microbial growth and enzyme expression, accelerating degradation in eutrophic waters and organic-rich sediments, whereas in oligotrophic systems, transformation is often slower. Consequently, microbial activity can reduce malathion’s environmental half-life from weeks to days in some systems, although the transient formation of toxic intermediates necessitates monitoring beyond the parent compound. Improved understanding of the microbial taxa, enzymatic machinery, and environmental drivers involved provides a stronger mechanistic framework for predicting malathion fate, refining ecological risk assessments, and informing targeted bioremediation strategies in contaminated ecosystems.

**Graphical Abstract:**

**Figure:**
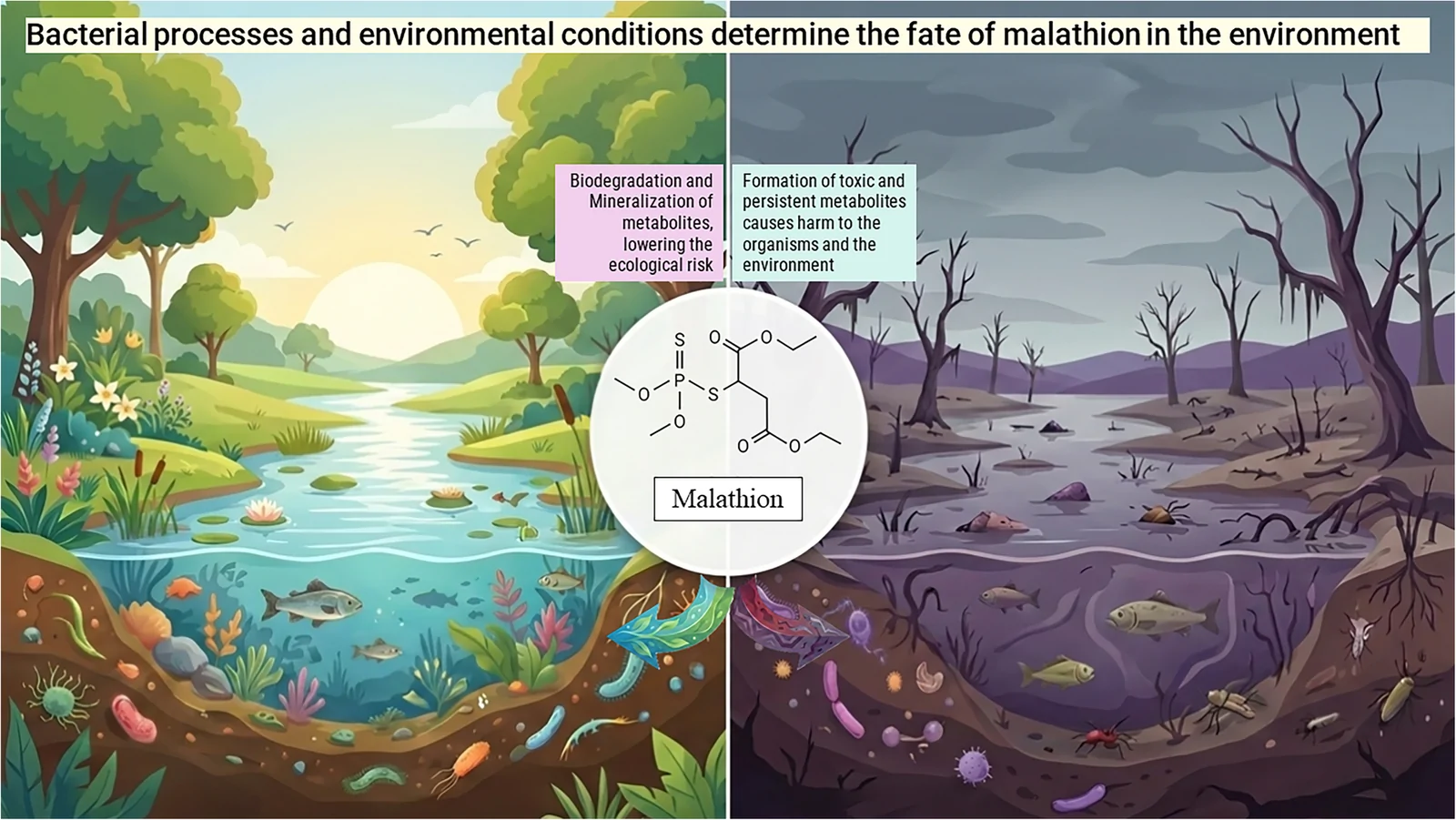

## Introduction

**Fig. 1:**
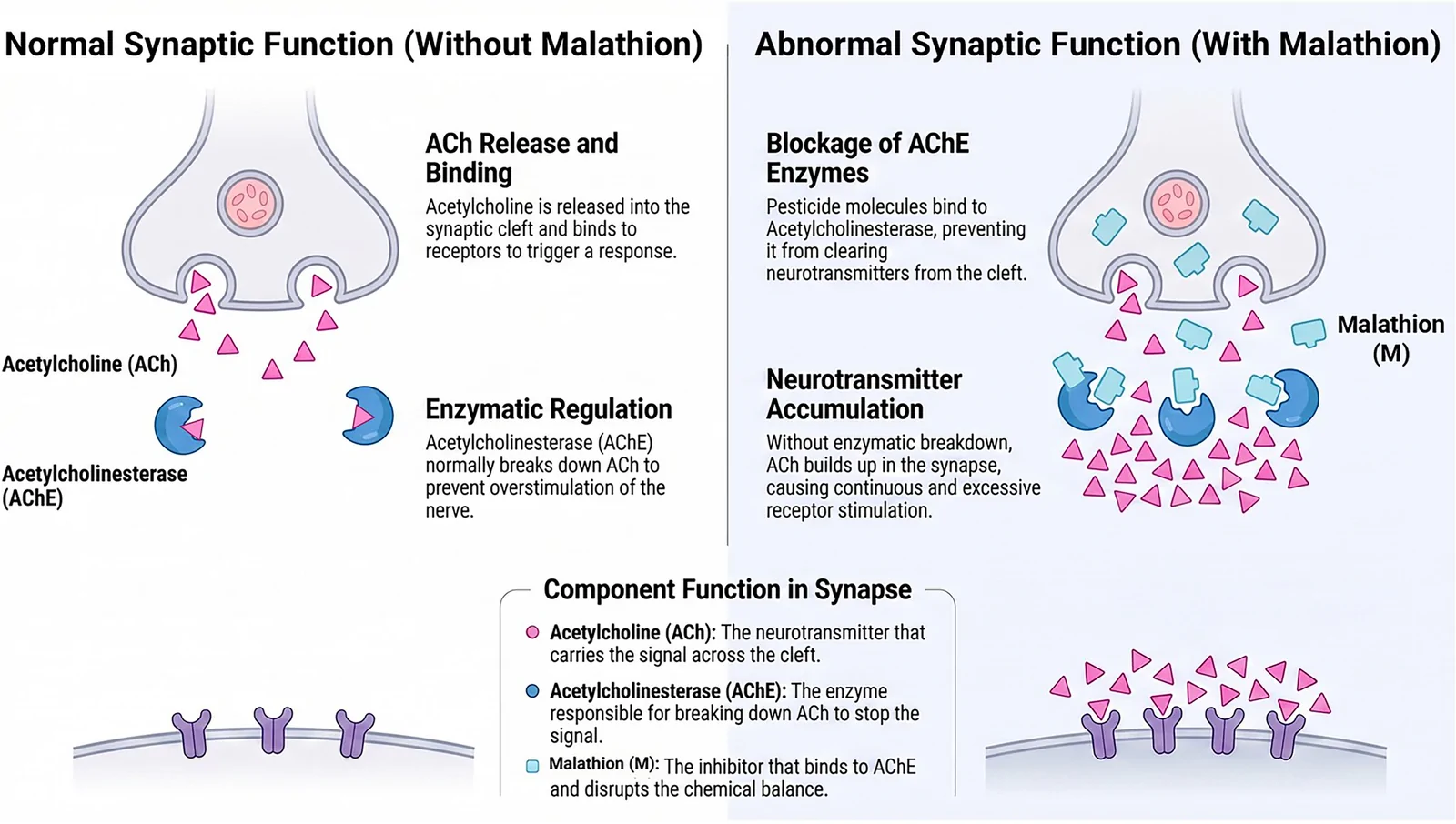
Schematic representation of the mechanism and inhibition of acetylcholinesterase enzyme in the presence and absence of malathion. Acetylcholine (ACh, pink) released into the synaptic cleft is hydrolysed by acetylcholinesterase (AChE, blue), terminating signal transmission. Malathion (M, light blue) binds to the AChE and blocks the hydrolysis, causing ACh to accumulate and stimulate postsynaptic receptors continuously. (Image generated by Google NotebookLM [Gemini model] based on the user-provided source documentation, Wang et al. 2025)

Malathion is a synthetic organophosphate insecticide classified chemically as an O, O-dimethyl dithiophosphate ester of diethyl mercaptosuccinate (CAS No. 121-75-5) (ATSDR, 2003; IARC 2017). Introduced in the 1950s, it gained widespread adoption for use in agricultural systems (e.g., fruits, vegetables, cereals, and cotton), forestry, ornamental plant management, livestock operations, large-scale mosquito abatement programs, golf courses, and stored products. It is also used in residential settings for garden pest control, flea treatment in pets, and head lice management in humans (ATSDR, 2003). The EPA has set the maximum residue limit of 8 ppm of malathion on most crops, including grapes, berries, citrus, tomato, pepper, potato, onion, and leafy vegetables (U.S. EPA 2025). Malathion inhibits the enzyme acetylcholinesterase in insects and in organisms exposed to it. The enzyme hydrolyses acetylcholine into choline and acetate, essential for terminating synaptic transmission and allowing the neuron to rest (Wang et al. 2025). This inhibition of the enzyme accumulates acetylcholine in the synaptic cleft, causing tremors, paralysis, and potential fatality in insects. The mechanism is represented in detail in Fig. 1 (IARC 2017; Kumar et al. 2019). Cytochrome P450 reactions in non-target organisms, catalyzed by monooxygenases, can transform malathion into malaoxon, which is significantly more toxic than its parent compound (Basapuram et al. 2025a; Wang et al. 2025). In terms of toxicity for humans, the World Health Organization has classified it to Group 2 A, potentially carcinogenic (ATSDR, 2003; IARC 2017; Patlolla et al. 2015).

Global use of malathion remains substantial despite the regulatory decline of many organophosphates in the early 2000s. Based on data synthesized from regulatory reports and global pesticide statistics, annual malathion production is estimated at several tens of thousands of metric tons (Gupta 2019). In the United States alone, historical usage has reached approximately 30 million pounds per year across both agricultural and non-agricultural sectors, with major applications in mosquito abatement programs in states such as California, Florida, and Texas (Yadav et al. 2023). In California, extensive aerial malathion spraying during fruit fly eradication programs in the late twentieth century involved the weekly dispersal of thousands of liters of malathion, illustrating the magnitude of environmental inputs (Gupta 2019). Globally, large-scale spraying campaigns remain common in Asia, Africa, and South America. For instance, India and China are among the largest consumers, using malathion primarily for crop protection (Van Dyk and Pletschke 2011). In sub-Saharan Africa, malathion has been widely employed in large-scale locust control operations, whereas in Latin America, it remains a major component of dengue and malaria vector control programs (Patlolla et al. 2015). Monitoring of Canadian watersheds has documented concentrations as high as 506 µg/L in streams affected by agricultural runoff, levels that exceed ecological safety thresholds (Richmond 2021). These data indicate that its widespread and intensive use results in its continual replenishment. Currently, 10 countries manufacture malathion, with major producers in China, India, the USA, the UK, Denmark, Egypt, Japan, Mexico, Singapore and Switzerland (ATSDR, 2003; Kumar et al. 2012; Richmond 2021; Yadav et al. 2023).

After application, malathion can enter the environment via spray deposition, atmospheric drift, leaching, and surface runoff (Duttagupta et al. 2020; Mukhopadhyay et al. 2022). Its relatively low soil sorption coefficient (K_oc_ ≈ 93–1800) favors partitioning into the water column rather than strong sorption to sediments (Van Dyk and Pletschke 2011). Reported concentrations range from roughly 55–75 µg/L in typical agricultural streams to > 500 µg/L in intensive-use areas (Singh 2008). In rainwater and fog, concentrations of 21–2740 ng/L demonstrate atmospheric transport and wet deposition (Gupta 2019; Stamm et al. 2025b). These levels are ecologically important because malathion is highly toxic to aquatic invertebrates and fish, with LC50 values generally in the low µg/L range (Ullah et al. 2018). Additional metabolites, including malathion mono- and dicarboxylic acids, also retain some biological activity (Van Dyk and Pletschke 2011). Therefore, environmental risk assessments should consider both the parent pesticide and its major transformation products. Malathion application rates are highly crop-specific. Average values of about 0.70 kg/ha for corn (maximum 1.12 kg/ha) and 1.68 kg/ha for cotton have been reported, while orchards and vineyards show a broader range (0.47–4.40 kg/ha, with a maximum of 8.42 kg/ha). For vegetables and ground fruits, applications typically range from 0.74 to 2.12 kg/ha, often approaching the U.S. maximum allowable rate of 2.24 kg/ha (Duttagupta et al. 2020; U.S. EPA, 2009). Consistent with these usage patterns, monitoring studies report strong seasonal variation in aquatic concentrations, with estimates of 88.11 µg/L in spring, 63.21 µg/L in winter, 28.44 µg/L in summer, and 8.58 µg/L during other times of the year (Gupta 2019; Richmond 2021; Singh 2008; Van Dyk and Pletschke 2011).

Historically, research on the fate of malathion has emphasized abiotic degradation processes such as hydrolysis and photolysis (Paschal and Neville 1976; Satish et al. 2017; Wang et al. 2025). However, bacterial biotransformation is a primary determinant of environmental persistence (Bollag et al. 1992; Bollag and Liu 1990; Kumar et al. 2019). Diverse bacterial communities in sediments and the water column express enzymes, including carboxylesterases, phosphotriesterases, and phosphatases, that catalyze hydrolytic cleavage of ester bonds, yielding mono- and dicarboxylic acids that may subsequently mineralize to carbon dioxide and inorganic phosphate under aerobic conditions (Basapuram et al. 2025b; Paschal and Neville 1976). In oxygen-limited environments, alternative reductive pathways can generate phosphorothionates and desmethylated intermediates (Hoagland et al. 2001; Van Eerd et al. 2003). Thus, community composition, redox status, temperature, and nutrient availability collectively regulate transformation trajectories and ecological outcomes. Given its global distribution, documented ecotoxicological effects, and the complexity of its transformation pathways, malathion represents a compelling model compound for integrating environmental chemistry with bacterial ecology. A comprehensive review of enzymatic mechanisms, bacterial taxa involved in degradation, and environmental controls on transformation rates is therefore essential. An integrated perspective can improve predictions of environmental persistence and inform risk assessment and bioremediation strategies in both aquatic and terrestrial systems.

## Toxicological and ecological effects of malathion exposure

The acute toxicity of malathion is dominated by cholinergic neurotoxicity after oxidative desulfuration of the phosphorothioate (P = S) group to form malaoxon (P = O). Malaoxon phosphorylates the catalytic serine of acetylcholinesterase (AChE), suppressing hydrolysis of acetylcholine and causing its accumulation at cholinergic synapses and neuromuscular junctions. The resulting overstimulation produces the muscarinic, nicotinic, and central nervous system manifestations characteristic of organophosphate poisoning. Because spontaneous reactivation becomes progressively less favorable after dealkylation (‘aging’) of the phosphorylated enzyme, the interaction is functionally irreversible on the physiological timescale. Regulatory assessments therefore use cholinesterase inhibition as a principal toxicological endpoint and apply an additional potency adjustment to malaoxon exposures (ATSDR, 2003; U.S. EPA, 2009). IARC has classified malathion as probably carcinogenic to humans (Group 2 A) based on limited evidence in humans, sufficient evidence in experimental animals, and mechanistic evidence (IARC 2017).

Experimental studies also identify non-cholinergic effects, although their strength and environmental relevance vary by endpoint and model. Malathion exposure has been associated with increased reactive oxygen species, lipid peroxidation, depletion or altered activity of antioxidant defenses, mitochondrial dysfunction, and oxidative injury in neural, hepatic, and branchial tissues. Genotoxic responses, including micronucleus formation and DNA strand damage, have been reported in fish exposed to sublethal concentrations. Reproductive and developmental studies in aquatic vertebrates describe reduced survival or growth, developmental abnormalities, gonadal injury, altered fertility, and changes in reproductive and thyroid-related hormones. These findings support consideration of oxidative, genomic, developmental, and endocrine endpoints in addition to acute mortality and AChE inhibition; however, they should not be interpreted as equally established across species or at all environmentally encountered concentrations.

Immunotoxicity has been demonstrated most consistently in mammalian experimental systems, where repeated malathion exposure altered antibody production, leukocyte function, macrophage responses, and other indices of humoral or cell-mediated immunity. Comparable environmentally relevant data for aquatic organisms remain limited. This evidence gap is important because immunological impairment could interact with infection, nutritional stress, and other contaminants in natural populations. Aquatic invertebrates are generally among the most sensitive non-target taxa because organophosphate exposure disrupts cholinergic signaling at low concentrations. Fish may also exhibit acute lethality and sublethal neurobehavioral, oxidative, developmental, and reproductive effects, with marked variation among species and life stages. U.S. EPA screening-level assessments have identified acute ecological risks of concern for fish and especially aquatic invertebrates under several registered-use scenarios (U.S. EPA, 2009). Ecological interpretation must therefore be transformation-product specific: carboxylesterase-mediated hydrolysis to malathion mono- and dicarboxylic acids generally reduces AChE-inhibitory potency, whereas oxidative desulfuration transiently produces malaoxon and can increase hazard before subsequent degradation. Monitoring parent-compound loss alone can consequently overestimate detoxification. Maximum residue limits (MRL) are legal, commodity-specific compliance values rather than toxicological thresholds, and direct numerical comparison requires attention to the residue definition used by each authority. For instance, U.S. tolerances under 40 CFR § 180.111 are generally specified for malathion but include malathion plus malaoxon for designated commodities, whereas the European Union database lists the applicable residue definition with each MRL. Codex maximum residue limits are international food-trade standards derived from supervised residue trials and dietary-risk assessment. Representative values are summarized in Tables 1 and 2.

**Table 1:** Representative regulatory residue limits for malathion

Regulatory authority	Representative entries (mg/kg)	Interpretive note
(U.S. EPA, 2009) / eCFR, 40 CFR § 180.111	Apple 8; grape 8; tomato 8; potato 8; egg 0.1; milk fat 0.5	Commodity-specific tolerances. Some designated commodities use the combined residues of malathion and malaoxon.
Codex Alimentarius (pesticide) (FAO, 2026)	Commodity-specific CXLs; consult the live Codex database	International standards; values and residue definitions are commodity-specific and may differ from national tolerances.
European Union pesticides database (EU, 2026)	Commodity-specific MRLs, frequently set at an analytical LOQ for unsupported uses	The database identifies values with an asterisk when set at the limit of quantification and provides the operative residue definition.

**Table 2:** Representative acute toxicity values. Values are formulation-, purity-, route-, duration-, and species-dependent; they are illustrative hazard endpoints, not environmental quality criteria. LD₅₀ denotes median lethal dose, LC₅₀ median lethal concentration, and EC₅₀ median effect concentration

Taxonomic group/test species	Acute endpoint	Interpretation/source
Mammal – rat	Oral LD₅₀: 5,400 mg/kg (male) and 5,700 mg/kg (female) in the acute technical-material study	Low acute oral toxicity of technical malathion in this guideline study (U.S. EPA, 2009).
Bird – ring-necked pheasant	8-day dietary LC₅₀: 2,369 mg/kg diet	Slightly toxic on an acute dietary basis; this is an LC₅₀, not an oral gavage LD₅₀ (U.S. EPA, 2009).
Fish – bluegill sunfish	96-h LC₅₀: 30 µg/L	Representative sensitive freshwater fish endpoint used in EPA ecological risk assessment (U.S. EPA, 2009).
Aquatic invertebrate – Daphnia magna	48-h EC₅₀: 1.0 µg/L	Representative sensitive freshwater invertebrate endpoint used in EPA ecological risk assessment (U.S. EPA, 2009).

## Bacterial enzymatic biotransformation of malathion

### Enzymatic mechanisms

**Fig. 2:**
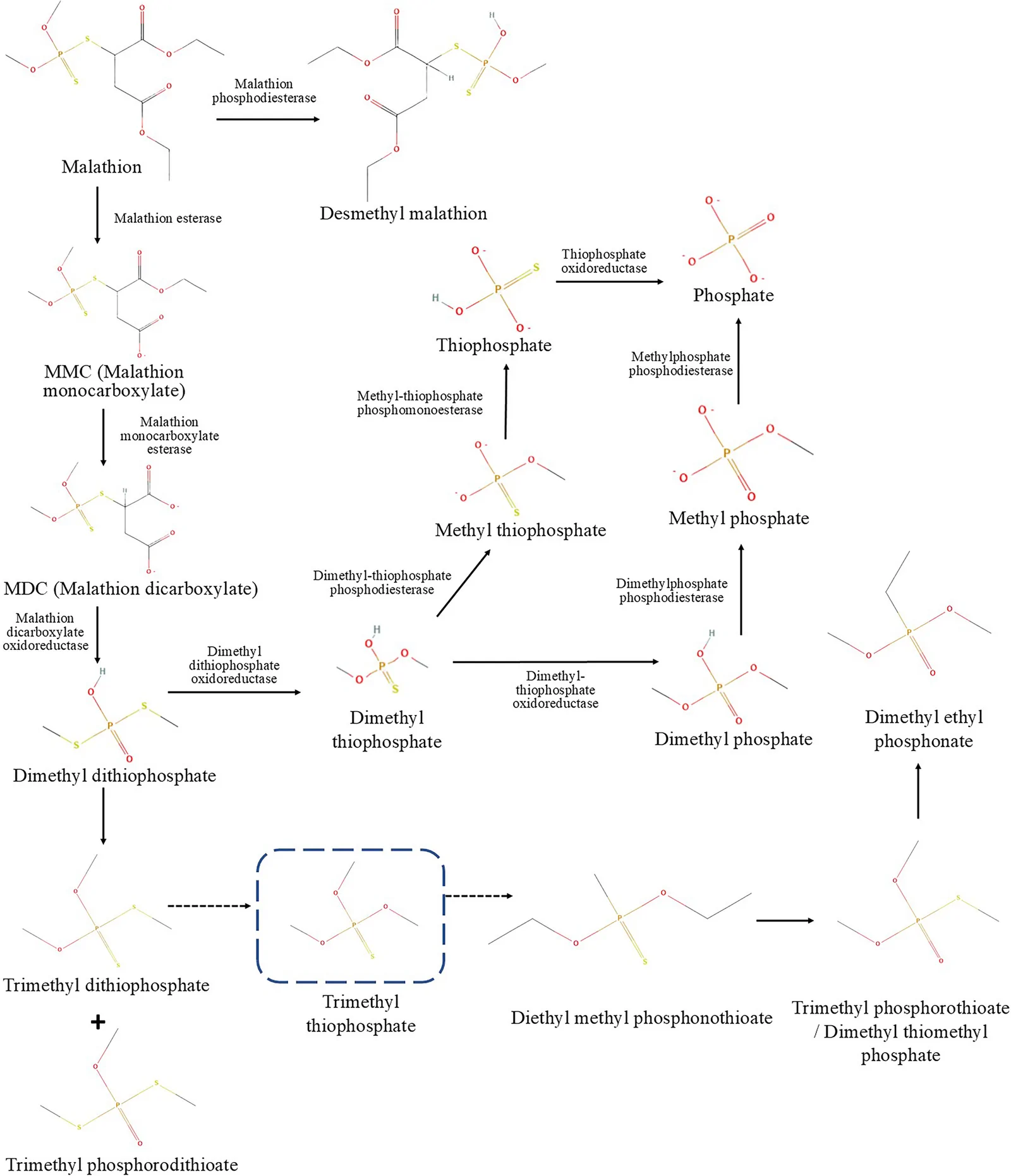
Proposed bacterial biotransformation pathway of malathion via esterase-mediated hydrolysis, showing major metabolites and associated enzymes. Some intermediates and enzymes have not been directly detected in bacterial isolates and are inferred based on previous reports; additionally, some metabolites may also form through abiotic transformation. The dotted arrow and dotted box indicate possible intermediate metabolites that were detected during malathion transformation; however, these intermediates have not been previously reported as part of the established pathway

**Fig. 3:**
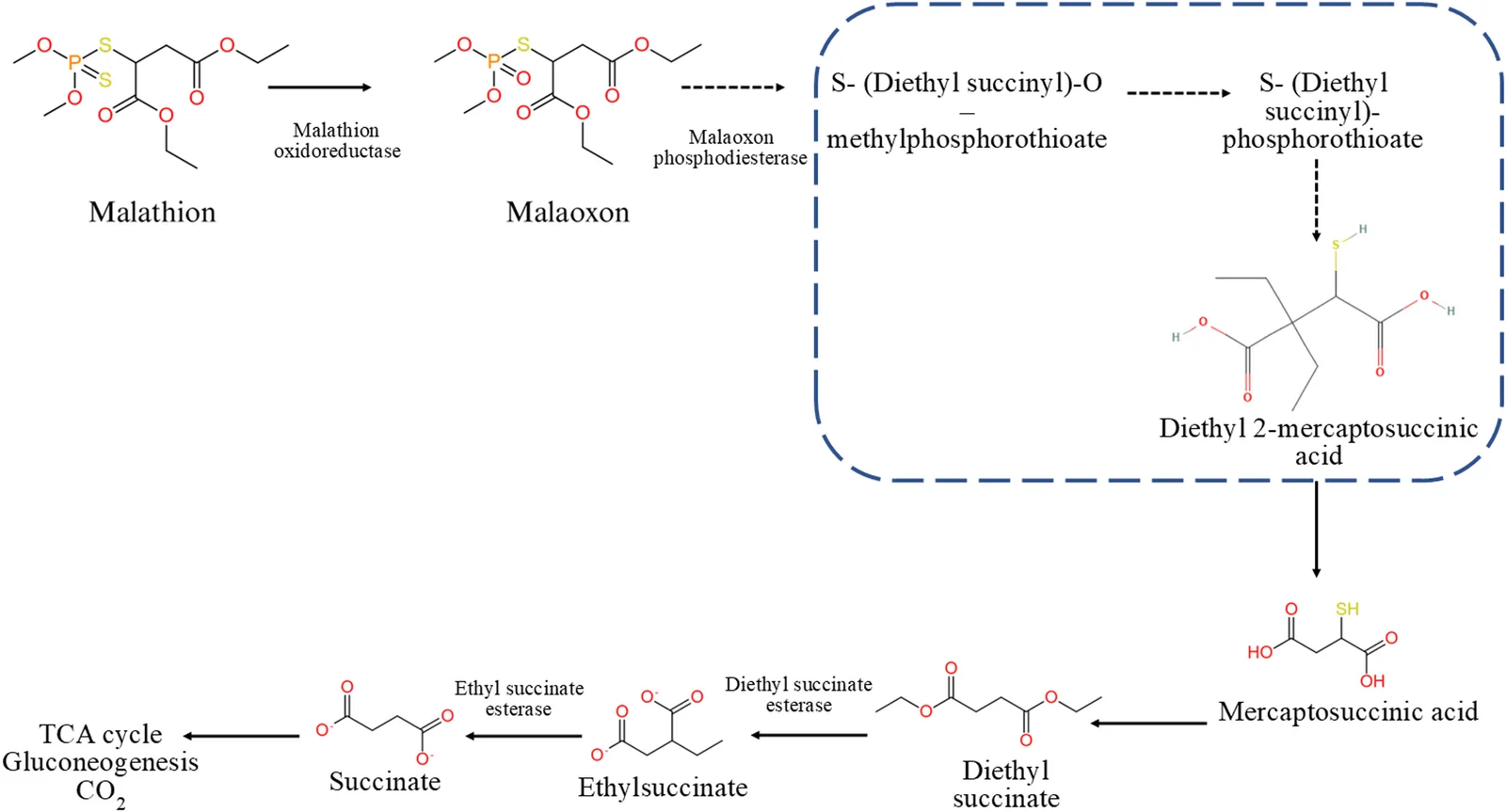
Proposed bacterial biotransformation pathway of malathion via oxidoreductase-mediated oxidative desulfuration, showing major metabolites and associated enzymes. Some intermediates and enzymes have not been directly detected in bacterial isolates and are inferred based on previous reports; additionally, some metabolites may also form through abiotic transformation. The dotted arrow and dotted box indicate possible intermediate metabolites that were detected during malathion transformation; however, these intermediates have not been previously reported as part of the established pathway

**Fig. 4:**
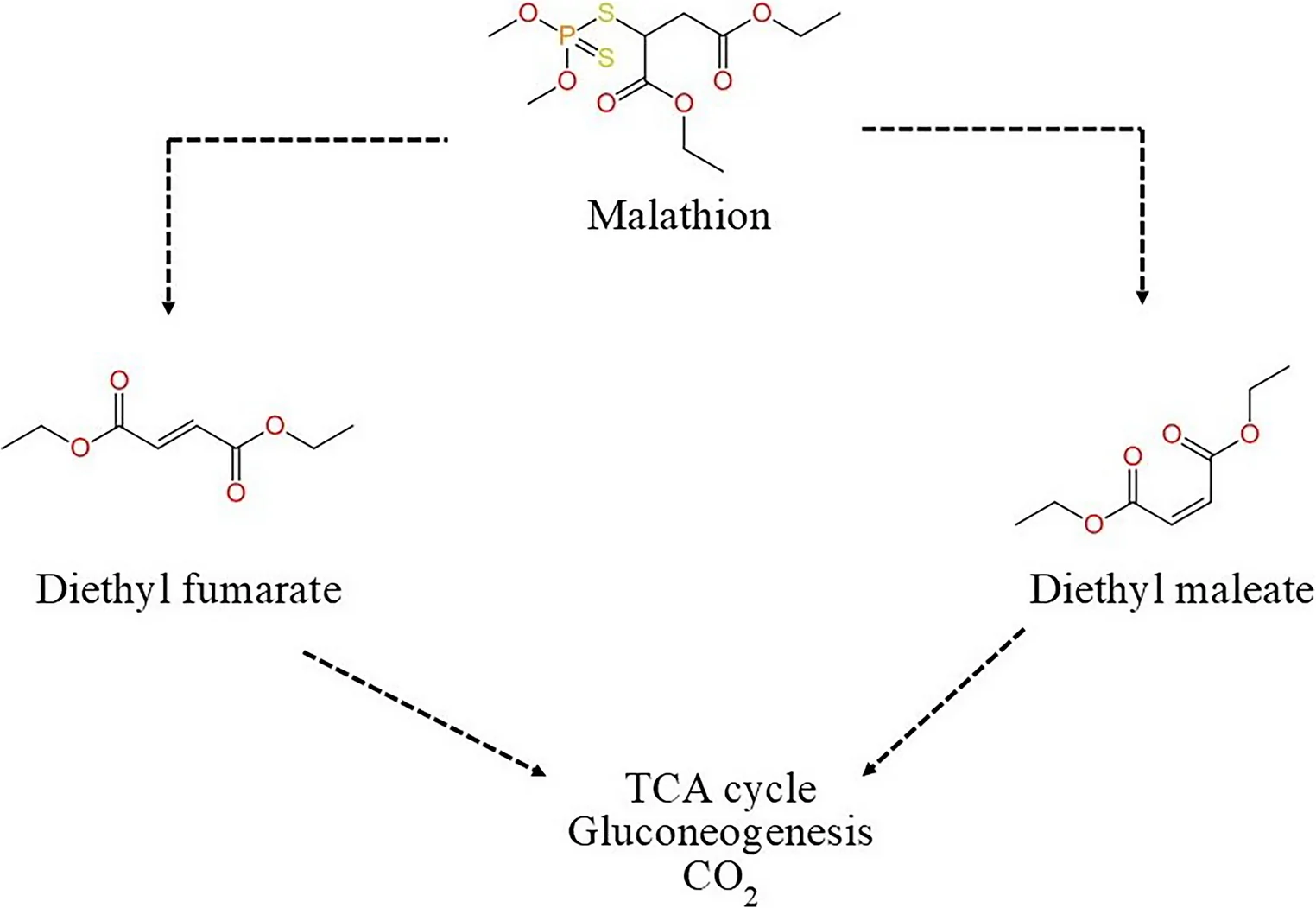
Proposed bacterial biotransformation pathway of malathion via hydrolysis. The intermediates and enzymes involved in the biotic conversion of malathion into diethyl fumarate and diethyl maleate have not been reported in previous literature. Diethyl maleate may also form through abiotic transformation. The dotted arrow and dotted box indicate possible intermediate metabolites detected during malathion transformation

Biotransformation is widely considered the predominant mechanism governing the environmental fate of malathion in sediments and aquatic systems. This degradation is fundamentally an enzyme-mediated process, and environmentally driven regulatory factors collectively determine transformation rates and metabolite profiles (Bollag et al. 1992; Bollag and Liu 1990). While abiotic hydrolysis and photolysis can contribute to malathion dissipation, substantial evidence indicates that enzymatic processes within bacterial communities are the primary determinant of malathion degradation in sediments, aquatic systems, and wastewater systems (Basapuram et al. 2024; Duttagupta et al. 2025; Racke et al. 1996; Singh 2008; Stamm et al. 2025a; Van Eerd et al. 2003). The principal route of malathion degradation involves enzymatic hydrolysis of its carboxylic ester linkages (Fig. 2). This process is mediated primarily by carboxylesterases, organophosphorus hydrolases, and related ester-cleaving enzymes. Another pathway of degradation involves oxidative transformation reactions (Fig. 3) that result in malaoxon formation (Anjum et al. 2022; Hoagland et al. 2001; Ishag et al. 2016; Kumar et al. 2019; Satish et al. 2017). Previous studies have also reported the formation of diethyl fumarate and diethyl maleate during malathion transformation (Verma et al. 2021; Dar and Kaushik 2023; Wang et al. 2025) (Fig. 4). However, the specific intermediates and enzymes involved in the biotic conversion of malathion to diethyl fumarate and diethyl maleate have not yet been reported. In addition, diethyl maleate may also be produced through abiotic transformation pathways (Wang et al. 2025).

The primary enzymatic mechanism responsible for malathion degradation is carboxylesterase-catalyzed hydrolysis (Fig. 2). This mechanism produces MMC and MDC, metabolites that reduce acetylcholinesterase inhibitory activity and are less toxic than the parent compound (Gupta and Gandhi 2023; Van Dyk and Pletschke 2011). Under aerobic conditions, these intermediates can be further mineralized by bacterial communities to carbon dioxide and inorganic phosphate (Satish et al. 2017; Sirajuddin et al. 2020). Early studies demonstrated that riverine bacterial consortia rapidly hydrolyze malathion, forming MMCα/β and MDC metabolites (Paris et al. 1975). Most recently, integrative genomic and metabolic profiling identified a suite of novel carboxylesterase and hydrolase gene clusters whose expression patterns during malathion exposure suggest previously uncharacterized catalytic intermediates; the findings demonstrate that even intensively studied degradation routes harbor mechanistic complexity not yet captured by conventional biochemical assays (Pillay et al. 2025a).

Studies on sediment/soil samples, specifically in agricultural and pesticide-polluted environments (Goda et al. 2010; Ishag et al. 2016), reported metabolite formation through carboxylesterase-mediated hydrolysis in aerobic carbon-limited conditions (Singh et al. 2012a, b) as well as oxic conditions (Thabit and El-Naggar 2013). Mechanistically, bacterial carboxylesterases attack the two ester carbonyl carbons of the diethyl succinate side chain rather than the central P–S or P–O linkages, proceeding via nucleophilic attack at the ester carbonyl carbon and cleavage of the alkyl–oxygen bond (Kumar et al. 2019). This sequentially releases the α- and β- MMC intermediates and ultimately MDC, leaving the phosphorus-bonded core of the molecule intact until later stages of mineralization.

In seawater, hydrolysis dominates the bacterial transformation pathway, with carboxylesterase activity producing MMC and MDC alongside malaoxon detected as a by-product, and microbial activity acting in parallel with sediment sorption to drive overall compound loss (Cotham Jr and Bidleman 1989). In wastewater and sewage matrices, the same carboxylesterase-mediated route prevails, with MMC and MDC again identified as the principal hydrolytic products (El-Sesy et al. 2026; Ewida et al. 2023; Sirajuddin et al. 2020) and degradation rates ranging from approximately 50% over 15 days (Mohamed et al. 2010) to 99% within 4 h under optimized conditions (Sirajuddin et al. 2020). Agricultural drainage water similarly supports hydrolytic degradation, with high-tolerance isolates achieving 70% removal of 700 mg/L malathion within 12 days (Abdelall et al. 2022). The mechanistic consistency across these different aquatic matrices indicates that the carboxylesterase route is robust to wide variations in salinity, organic loading, and ionic composition. Together, these findings underscore the central role of carboxylesterases as functional biomarkers of malathion biotransformation capacity (Gupta 2019). More recent investigations have broadened the understanding of bacterial degradation capacity under extreme environmental conditions.

An alternative enzymatic route involves oxidative desulfuration of the thiophosphate (P = S) moiety, yielding malaoxon (Paschal and Neville 1976). The reaction is catalyzed by monooxygenase–type oxidoreductases that replace sulfur with an oxygen atom (Fig. 3), replacing the sulfur (Health Canada 2023; U.S. EPA, 2009). This pathway involves further degradation of malaoxon through phosphodiesterase and phosphatase, releasing diethyl succinate and succinate as end products.

### Bacterial taxa Capable of Malathion Transformation

The phylogenetic extent of malathion- transforming bacteria has expanded considerably with each new study of previously unexplored environments. Table 3 summarizes representative isolates, their environmental origins, and probable metabolic pathways.

**Table 3:** List of studies on bacterial biotransformation pathways and metabolites, along with study location

Microorganism	Matrix / Source	Probable Metabolic Pathway	Malathion Concentration and Condition	Degradation percentage	Location	Study
20 isolates were tested	Sediment and water samples from salt marshes	Carboxylesterase-mediated hydrolysis; MMC, MDC^&^	100 mg/L; 28 ℃	NR	Florida, USA	(Bourquin 1977)
5 Bacterial isolates (enriched)	Reclaimed Agricultural soil	Carboxylesterase-mediated hydrolysis; MMC, MDC^&^	5 µg/mL; pH 7.3 ± 0.1; 33 ± 2 °C	NR	Egypt	(Thabit and El-Naggar 2013)
*Acinetobacter baumannii* AFA	Domestic sewage - culture	Hydrolysis	100 mg/L; 30 ℃; pH 7–7.5	84% in 14 days	Egypt	(Azmy 2015)
*Acinetobacter johnsonii* MA19	Aquatic isolates/lab medium	unspecified	100 mg/L; 30 ℃; pH 7.0–7.5	100% in 84 h	Beijing, China	(Shan et al. 2009)
Algae–bacteria consortia	Aerated/non-aerated water	Mineralization to P_i_	NR	NR	China	(Wang et al. 2023)
*Alicyclobacillus tengchongensis*	Lab culture	Carboxylesterase-mediated hydrolysis; MMC, MDC^&^	100 mg/L; pH 7; 37 ℃	50% in 25 min and 89% in 100 min	China	(Xie et al. 2013)
*Alphaprotoebacterium* BAL284 and *Pseudomonas putida* 32zhy	Agricultural soil	Carboxylesterase-mediated hydrolysis; MMC, MDC^&^	50 mg/L; 30 ℃	43% in 2 days	Egypt	(Goda et al. 2010)
*Arthrobacter* species	Sediment	Esterase-driven hydrolysis^&^	NR	97%	Mississippi, USA	(Walker and Stojanovic 1974)
*Bacillus licheniformis* ML-1	Soil/sediment isolates	Hydrolysis	25 ppm; 32 ℃; pH 7.5	87% in 96 h	Pakistan	(Khan et al. 2016)
*Bacillus safensis* FO-36bT, *Bacillus subtilis* subsp. *inaquosorum* KCTC13429T, and *Bacillus cereus* ATCC14579T	Pesticide-polluted soil	No metabolites detected	400 mg/L; 25 ℃	NR	Sudan	(Ishag et al. 2016)
*Bacillus* sp. AGM5	Soil/sediment isolate - aqueous	Hydrolysis	300 µl/L and 500 µl/L; pH 7 and 8; 35 ℃ and 40 ℃	72.5% in 360 h	Rajasthan, India	(Dar and Kaushik 2022)
*Bacillus* sp. FYM31	Agricultural drainage water	Hydrolysis	700 mg/L, 37 °C	70.1% in 12 days	Egypt	(Abdelall et al. 2022)
*Bacillus thuringiensis* MOS-5	Wastewater	Hydrolysis	250 ppm, pH 5 and 7; 30 ℃ and 50 ℃; NaCl – 1, 5, and 7%	91% in 15 days	Egypt	(Mohamed et al. 2010)
Bacteria + fungi	Water/soil isolates	Hydrolysis/cometabolism	NR	NR	Egypt	(Ewida et al. 2023)
Bacterial consortia (enriched)	River water isolates	Hydrolysis; MMCα/β, MDC^&^	NR	NR	Georgia, USA	(Paris et al. 1975)
*Brevibacillus* KB2; *Bacillus cereus* PU	Soil/sediment enrichments	MMC, MDC^&^	0.05% − 0.2%; 30 ℃	NR	Punjab, India	(Singh et al. 2012a)
*Brevibacillus* KB2; *B. cereus* PU	As above	Hydrolysis	0.05% − 0.2%; 30 ℃	36.22% and 49.31% in 7 days	Punjab, India	(Singh et al. 2012a)
*Escherichia coli* IES-02	Wastewater	Carboxylesterase-mediated hydrolysis; MMC, MDC^&^	0.1, 0.5, 2, 50 ppm; 37 ℃	81% in 20 min	Pakistan	(Sirajuddin et al. 2020)
*Lysinibacillus* sp.	Soil	Transformation; mono/di-acids^&^	0.15%; 30 ℃	20% in 7 days	Punjab, India	(Singh et al. 2012b)
*Micrococcus aloeverae* MAGK3 *+ Bacillus cereus* AGB3 *+ Bacillus paramycoides* AGM5	Soil/Sediment	Oxidative Desulfuration: Malaoxon and Carboxylesterase-mediated hydrolysis; MMC, MDC^&, #, and $^	500 mg/kg	76.58%, 70.95%, and 88.61%	Rajasthan, India	(Dar and Kaushik 2023)
Natural consortia	Seawater & seawater–sediment microcosms	Biotic + abiotic; hydrolysis dominates; malaoxon observed^#^	Seawater- 122 ng/ml, 20 ℃, pH 8.0-8.2 Seawater-sediment – 3669 ng/mg, 20 ℃, pH 7.3–7.7	NR	South Carolina, USA	(Cotham Jr and Bidleman 1989)
*Ochrobactrum* sp. M1D	Cold water matrices	Psychrotolerant CE activity and Hydrolysis at low Temperature^&, #, and $^	100 mg/L, (15, 20, 28, 37) ℃, pH 6, 7, 8	100% in 12 days	Western Himalayan region, India	(Verma et al. 2021)
*Pseudidiomarina homiensis* FG2; *Pseudidiomarina* sp. CB1; other deep-sea isolates	Deep-sea hydrothermal sediment - culture	CE-mediated MMCα/β; MDC^&^	500 mg/L, 28 ℃, pH 7	100% in 36 h	China	(Ma et al. 2022)
*Pseudomonas putida*	Agricultural wastewater	Carboxylesterase-mediated hydrolysis; MMC, MDC^&^	50, 100, 150 mg/L; pH 5, 7, 9	93.81%	Egypt	(El-Sesy et al. 2026)
*Pseudomonas putida*,* Rhodococcus rhodochrous*, and *Sphingomonas* sp.	Aquatic lab media	Esterase-driven hydrolysis^&^	25, 50, 75, 100, 125, 150, 175 and 200 mg/L, 30 ℃	72%	Varanasi, India	(Geed et al. 2016)
*Pseudomonas stutzeri* MTCC-2643	Aquatic reactors	Hydrolysis	50, 100, 150, 200 mg/L, 37 ℃	150 ppm in 30 h	Tamil Nadu, India	(Vaishali et al. 2020)
*Rhodococcus tibetensis* sp. nov.	Soil/sediment	No metabolites detected	100 mg/L, 10 ℃ or 24 ℃	99.9%	Tibet, China	(Huang et al. 2022)
*Rossellomorea* sp.	River water	Oxidative desulfuration^#^	1%; pH 5, 7, 9; (25, 30, 35) ℃	NR	Georgia, USA	(Pillay et al. 2025a)
*Shewanella oneidensis*	Lab culture	No metabolites detected	10, 20, 30 mg/L; pH 4, 5, 6, 7; 35 ℃	84.1, 91.6, and 94.0% in 7 days	China	(Pan et al. 2024)

N.B.: ‘&’ denotes the pathway shown in Fig. 2; ‘#’ denotes the pathway shown in Fig. 3; and ‘$’ denotes the pathway shown in Fig. 4. *CE-* carboxylesterase activity, *MMC-* malathion monocarboxylic acid, *MDC*- malathion dicarboxylic acid. *NR-* Not reported

Within the phylum Firmicutes, species of *Bacillus* have received the greatest attention, probably because they form heat- and desiccation-resistant endospores that persist through agricultural storage and soil drying cycles, allowing easy isolation by culture-based methods. *Bacillus licheniformis* ML-1, isolated from agricultural soils, demonstrated 78% mineralization of environmentally relevant malathion concentrations (10–350 µg/L) within approximately five days, with cell-free esterase extracts retaining activity across a wide pH and temperature range (Khan et al. 2016). *Bacillus cereus* PU and *Brevibacillus* sp. KB2, recovered from Indian Punjab soils, degraded malathion under carbon-limited aerobic conditions at concentrations up to 0.15% (w/v) and notably exhibited even faster turnover of malaoxon than of the parent compound, an observation with important risk implications because it suggests that transient malaoxon produced in situ would be rapidly cleared in the presence of these organisms (Singh et al. 2012a, b, 2014). *Bacillus thuringiensis* MOS-5 isolated from Egyptian wastewater achieved approximately 50% degradation over 15 days, confirming that wastewater strains from high-nutrient aquatic matrices can also contribute to malathion removal (Mohamed et al. 2010). Multi-strain consortia of *Bacillus paramycoides*, *B. cereus*, and *Micrococcus aloeverae* outperformed any single constituent, reaching 88% degradation of 500 mg/kg malathion in amended soil within 15 days (Dar and Kaushik 2023).

Among the Pseudomonadota, *Pseudomonas* species represent the most intensively studied genus, reflecting their ubiquity in aerobic sediments and their well-documented capacity for malathion degradation. *Pseudomonas stutzeri* MTCC-2643 sustained reliable removal in aquatic reactor experiments, while *Pseudomonas aeruginosa* S1 achieved 33–48% degradation from 5 µg/mL initial concentrations in reclaimed agricultural soil, conditions that approach field-measured residue levels (Thabit and El-Naggar 2013; Vaishali et al. 2020). An isolate such as *Escherichia coli* IES-02 can degrade up to 99% of malathion within 4 h (Sirajuddin et al. 2020). The genus *Acinetobacter* offers additional diversity: *A. johnsonii* MA19 from Beijing showed that co-metabolic substrates could substantially accelerate aquatic degradation rates (Shan et al. 2009). *Brevibacillus* KB2, *Bacillus cereus* PU (Singh et al. 2012a), and *Lysinibacillus* sp. (Singh et al. 2012b) have demonstrated efficient hydrolysis of malathion under oxic laboratory conditions. Across these studies, MMC and MDC consistently appear as primary intermediates. *A. baumannii* AFA isolated from Egyptian domestic sewage degraded malathion rapidly in liquid culture, identifying wastewater infrastructure as a reservoir of active degraders relevant to effluent management (Azmy 2015).

Multi-compound degradation studies conducted with strains isolated from pesticide-polluted soils in Sudan further demonstrated that *Pseudomonas* and *Bacillus* isolates capable of degrading malathion could simultaneously transform chlorpyrifos and dimethoate, confirming that the catalytic breadth of organophosphate-hydrolyzing enzymes in these genera extends well beyond any single substrate (Ishag et al. 2016). *Ochrobactrum* sp. M1D, a psychrotolerant from Himalayan peach orchards, stands out for its capacity to achieve 100% degradation of 100 mg/L malathion within 12 days with confirmed metabolite profiles showing methyl phosphate, diethyl maleate, and diethyl 2-mercaptosuccinate as primary products (Verma et al. 2021). Similarly, agricultural drainage isolates removed approximately 70% of 700 mg/L malathion within 12 days (Abdellal et al. 2022), demonstrating substantial tolerance and degradation potential in contaminated matrices.

A previous study conducted a comparative analysis of bacterial isolates, revealing that the higher capacity of malathion degradation through oxidative desulfuration and carboxylic hydrolysis was achieved by *Pseudomonas putida* (72% by day 7 – phylum Pseudomonadota), *Sphingomonas* sp. (64% by day 9 – phylum Pseudomonadota), followed by *Rhodococcus rhodochrous* (70% by day 8 – phylum Actinomycetota) (Geed et al. 2016). The study also reported an increase in dry cell mass during incubation, from which it can be hypothesized that malathion might serve as a carbon source (Pillay et al. 2025b). The novel Actinobacterium *Rhodococcus tibetensis* sp. nov. FXJ9.536 (Huang et al. 2022), isolated from Qinghai-Tibet Plateau soil, degraded 99.85% of malathion at 24 °C and 50% at 10 °C; genome annotation identified a putative carboxylesterase (peg. 1365) sharing 31.5% amino acid identity with a characterized malathion-hydrolyzing carboxylesterase from the thermophile *Alicyclobacillus tengchongensis* of the phylum Bacillota. The enzyme was cloned and expressed in *Escherichia coli*, with optimal activity at 60 °C and pH 7 with β–naphthyl acetate, and a capacity to hydrolyze 89% of 5 mg/L malathion within 100 min (Xie et al. 2013).

Deep-sea hydrothermal sediment isolates of the class Gammaproteobacteria - *Pseudidiomarina* and *Idiomarina* achieved complete removal of 500 mg/L malathion within 36 h, demonstrating exceptional enzymatic efficiency. Genomic analyses of these strains identified multiple carboxylesterase genes associated with MMC and MDC formation (Ma et al. 2022). Other bacterial consortia showed 32–57% removal within 48 h, highlighting marked inter-strain variability (Wang et al. 2025). These findings confirm that marine sediments can sustain active malathion biotransformation even in the absence of prior pesticide exposure, and that deep-sea bacterial communities may contribute meaningfully to attenuating malathion transported to ocean sinks via runoff. The isolated strain *Shewanella oneidensis* MR-1, valued for its electron-donor capacity and broad metabolic flexibility, achieved 84.1%, 91.6%, and 94.0% removal of malathion over 7 days at initial concentrations of 10, 20, and 30 mg/L, respectively, extending the taxonomic range of confirmed degraders into a genus better known for its role in metal and azo-dye reduction (Pan et al. 2024).

Salt-marsh environments represent a third category of aquatically relevant sediment systems studied early in the field’s history. A previous study reported the isolation of 20 bacterial strains from Florida salt-marsh water and sediment, of which 11 could use malathion as a sole carbon source and 4 required peptone supplementation for co-metabolism (Bourquin 1977). Degradation efficiencies ranged from 48 to 90%, with degradation products limited to MMC and MDC, notably without detectable malaoxon, a finding consistent with predominance of carboxylesterase over monooxygenase activity in those organisms (Newhart 2006; Wolfe et al. 1975). Arthrobacter sp. isolates from Mississippi alluvial sediments similarly transformed 47–95% of applied malathion, generating an array of phosphorothioate and phosphorodithioate products en route to mineralization (Walker and Stojanovic 1974).

### Genes Involved in Malathion Degradation

The microbial degradation of malathion proceeds through breakage at two chemically distinct bonds: the two carboxylic-ester bonds and the central phosphorothioate (P-S) linkage. Based on this, the degradative genes are known to encode enzymes of two principal classes. The most frequently reported are the carboxylesterases, which hydrolyze the ester bonds to yield the less toxic malathion MMC and MDC. A study PCR-amplified a carboxylesterase gene from the genome of *Pseudomonas putida* IS2 whose sequence aligned closely with previously described *Pseudomonas* esterases (Goda et al. 2010). Another study amplified and sequenced a carboxylesterase gene fragment from *Acinetobacter baumannii* AFA (Azmy 2015). Among Gram-positive isolates, a putative malathion-degrading carboxylesterase gene was cloned and sequenced from *Brevibacillus* sp. KB2 and *Bacillus cereus* PU using a primer-based PCR strategy (Singh et al. 2012a). Another study characterized a malathion carboxylesterase from the crude extract of *Bacillus licheniformis* ML-1 (Khan et al. 2016), whereas a study by Sirajuddin et al. (2020) purified a novel multi-band carboxylesterase (≈ 33/30/28 kDa) from *Escherichia coli* IES-02. Ma et al. (2022) reported that the draft genomes of deep-sea *Pseudidiomarina* strains FG2/CB1 were abundant in carboxylesterase genes and degrade through the MMC and MDC pathway. Several of these enzymes have been characterized at the residue level and engineered for enhanced stability. A study by Xie et al. (2013) cloned the carboxylesterase gene *D1CarE5* from *Alicyclobacillus tengchongensis* and expressed it in *E.coli*, describing a 513-amino acid, ≈ 57.8 kDa enzyme bearing a Ser-Glu-His catalytic triad, while Yang et al. (2020) cloned the carboxylesterase gene *est741* from *Geobacillus uzenensis*, immobilized the resulting enzyme, and generated the improved mutant EstM160K.

The second class comprises the organophosphorus hydrolases (OPH), encoded by *opd*, which act directly on the organophosphate core, as reported for *Bacillus* sp. FYM31 (Abdelall et al. 2022). Genome-resolved studies have linked these activities to specific genes. In *Modestobacter marinus* G96, a putative malathion-degradation pathway was reconstructed involving a carboxylesterase, a cytochrome P450 monooxygenase, and an alkaline phosphatase, with the genes encoding these enzymes distributed across the genome (Hajri et al. 2026).

Native OPH hydrolyzes P-O bonds efficiently but acts only weakly on the P-S linkage of malathion, a limitation directly addressed by Schofield and DiNovo (2010). Through saturation mutagenesis at selected active-site codons of the *opd*-encoded enzyme, they generated variants with markedly improved P-S hydrolysis. In *Escherichia coli* lysates, expressing these variants exhibited up to 1800-fold higher specific activity toward malathion than wild-type lysates. The purified variant enzymes improved the specificity constant (*k*cat/*K*m) up to 25-fold for both substrates. Loss of acetylcholinesterase inhibition by the reaction products confirmed detoxification. This study represents the first study to report catalytic-efficiency data for a malathion-degrading enzyme, underscoring the potential of protein engineering to enhance the enzymatic bioremediation of malathion.

### Comparison of free enzymes, immobilized biocatalysts, and microbial consortia

The choice of biocatalyst formulation (free enzymes, immobilized biocatalysts, and microbial consortia) strongly influences malathion degradation efficiency and application. Free enzymes enable direct kinetic and mechanistic characterization but may have limited stability and reusability. Enzyme immobilization can improve recovery, operational stability, and continuous-flow performance, although diffusion limitations and unfavorable enzyme orientation may reduce apparent activity (Yang et al. 2020). Immobilized cells can support cofactor regeneration and multistep metabolism but may be constrained by mass transfer, nutrient availability, and biomass instability; moreover, malathion-specific evidence remains limited. Microbial consortia can combine complementary pathways and promote downstream metabolite transformation, as demonstrated by the improved performance of mixed *Bacillus–Micrococcus* cultures (Dar and Kaushik 2023), but their composition and activity may be difficult to control. Thus, free enzymes are most appropriate for controlled single-step conversion, whereas immobilized biocatalysts and microbial consortia may be more suitable for reusable treatment systems and complex environmental matrices, respectively. Malathion-specific experimental evidence for immobilized whole-cell systems remains limited; therefore, their proposed applications are based partly on broader principles of microbial biocatalysis (Table 4).

**Table 4:** Comparison of biocatalyst formats used or proposed for malathion degradation and bioremediation

Format	Principal advantages	Principal limitations and most appropriate use
Free enzyme	Enables direct measurement of K_m_*, k_cat_*, and k_cat_/K_m_, minimizes cellular mass-transfer limitations, and supports product-resolved mechanistic analysis. Engineered organophosphorus hydrolase variants showed enhanced catalytic activity toward malathion relative to the wild-type enzyme (Schofield and DiNovo 2010).	Limited operational lifetime and reusability and potential sensitivity to pH, temperature, inhibitors, and proteolysis; most appropriate for mechanistic studies and treatment of chemically defined streams.
Immobilized enzyme	Facilitates enzyme recovery and reuse, can improve operational stability, and is compatible with continuous-flow treatment. Immobilized Est741 carboxylesterase was evaluated for malathion and pyrethroid degradation in a packed-bed reactor (Yang et al. 2020).	Immobilization may reduce apparent activity through diffusion resistance, restricted conformational mobility, pore blockage, or unfavorable enzyme orientation; most appropriate when repeated catalyst use offsets immobilization and support costs.
Immobilized microbial cells	Retains biomass and whole-cell metabolic pathways and permits intracellular cofactor regeneration, potentially supporting sequential transformation reactions (Cassidy et al. 1996; Kourkoutas et al. 2004).	Substrate, oxygen, and nutrient transfer limitations, carrier instability, biomass decay, and cell leakage may constrain performance. Malathion-specific experimental evidence remains limited; therefore, this should be considered a prospective treatment approach.
Microbial consortium	Provides complementary enzymatic pathways, metabolic cross-feeding, and functional redundancy. A consortium of *Bacillus paramycoides*, *Bacillus cereus*, and *Micrococcus aloeverae* removed more malathion than the corresponding monocultures (Dar and Kaushik 2023).	Community drift, competition, antagonism, and invasion by indigenous microorganisms can reduce reproducibility and process control. Parent-compound disappearance should not be interpreted as complete detoxification without measurement of malaoxon, other transformation products, mineralization, or residual toxicity.

* K_m_, Michaelis constant; k_cat_, catalytic turnover number

### AI-assisted discovery and engineering of malathion-degrading enzymes

Metagenomic and metatranscriptomic datasets can be screened for divergent carboxylesterases and organophosphorus hydrolases using profile hidden Markov models, sequence-similarity networks, genomic context, and exposure-responsive expression data (Gerlt et al. 2015). Protein structures predicted with AlphaFold-class methods can then guide identification of putative catalytic residues and selection of sites for focused mutagenesis (Abramson et al. 2024; Jumper et al. 2021). Docking and molecular-dynamics simulations may help prioritize enzyme–substrate interactions involving malathion and malaoxon, but predicted binding does not demonstrate catalysis and can be affected by uncertainty in active-site conformation, protonation, and induced fit (Wong et al. 2022). Machine-learning-guided directed evolution may further reduce the experimental search space by prioritizing mutations associated with improved activity, stability, or substrate specificity (Yang et al. 2019). Nevertheless, computational predictions require biochemical validation through product identification and kinetic measurements. For malathion bioremediation, validation should additionally include malaoxon quantification and residual acetylcholinesterase-inhibition assays because enhanced parent-compound removal does not necessarily demonstrate detoxification.

## Environmental controls on degradation

### Temperature

Temperature is a major determinant of the rate of microbial malathion degradation. This relationship was reported early in riverine bacterial consortia (Paris et al. 1975) and has since been observed in isolates with mesophilic, thermotolerant, and psychrotolerant traits. This pattern is consistent with the rates of enzyme-mediated hydrolysis, which increase within the mesophilic range alongside bacterial growth (Dar and Kaushik 2022; Paris et al. 1975). The cell-free esterase of *Bacillus licheniformis* ML-1 has been noted for activity across a relatively broad temperature range, which may be advantageous for applications in environments with fluctuating thermal conditions (Khan et al. 2016). In applied terms, studies commonly report faster malathion degradation under warm conditions. For example, *Bacillus* sp. FYM31, isolated from Egyptian agricultural drainage, removed 70% of 700 mg/L malathion within 12 days at 37 °C, indicating both substantial concentration tolerance and effective degradation at elevated temperature (Abdelall et al. 2022). Similarly, *Micrococcus aloeverae* MAGK3 degraded 69.84% of 70 mg/L malathion within 24 h at 40 °C, suggesting that high summer temperatures in shallow agricultural waters may still permit, and in some cases enhance, degradation by thermotolerant strains (Dar and Kaushik 2023).

At lower temperatures, *Ochrobactrum* sp. M1D from the western Himalayan region hydrolyzed malathion under cold-water conditions that would likely suppress the activity of many mesophilic isolates, demonstrating that carboxylesterase-mediated transformation can also proceed in low-temperature waters and sediments (Verma et al. 2021). *Rhodococcus tibetensis* FXJ9.536 from Qinghai-Tibet Plateau soil retained 50% degradation activity at 10 °C, with genome analysis pointing to cold-shock proteins homologous to a malathion-degrading enzyme as the molecular basis for cold-adapted activity (Huang et al. 2022). Although cold-adapted malathion degradation has been reported in many earlier studies focused on temperate and tropical agricultural settings, these findings suggest that such activity may also be relevant in temperate winter waters, alpine lakes, and high-latitude reservoirs (Al-Wabel et al. 2010; Ligaray et al. 2017).

### pH

pH is an important control on bacterial malathion degradation, with most studies indicating the fastest rates under neutral to mildly alkaline conditions, typically around pH 7–8. Supporting this pattern, the cell-free esterase from *Bacillus licheniformis* ML-1 remained active at a pH range of 4–10 (with optimal activity at 7.5 and stable activity at 9) and was proposed to be suitable for chemically variable aquatic environments (Khan et al. 2016). More direct quantitative evidence comes from optimization studies: in a Taguchi experimental design, *Bacillus* sp. AGM5 showed that pH was among the most influential variables, and that optimized conditions yielded substantially greater degradation than unoptimized baselines, with the highest activity at neutral to mildly alkaline pH (Dar and Kaushik 2022). This corroborates carboxylesterase-mediated hydrolysis being a major route of bacterial transformation. Evidence from marine systems supports the combined importance of abiotic and biotic removal (Freitas et al. 2012). In seawater microcosms at pH 8.0, the half-life of malathion was approximately 2.6 days in unsterilized seawater, whereas sterilized controls exhibited nearly twofold longer half-lives of 5.3 days (Cotham Jr and Bidleman 1989). This pattern indicates that chemical hydrolysis at marine pH is significant and that microbial processes further accelerate dissipation rather than being masked by abiotic loss (Wang and Hoffman 1991).

### Redox conditions

Redox state affects both how malathion is transformed and which microbial communities are likely to dominate during its degradation. In aerobic systems, hydrolytic transformation is commonly the main pathway, consistent with the activity of carboxylesterase-producing bacteria and with the greater capacity for further oxidation and mineralization of hydrolysis products when oxygen is available (ATSDR, 2003). As a result, oxic surface waters, aerated sediment surfaces, and engineered systems with active aeration often show faster malathion loss, while intermediates such as MMC and MDC may accumulate only briefly before further degradation. In oxygen-depleted sediment zones, malathion transformation may therefore shift toward other pathways, including hydrolytic and reductive reactions that can generate phosphorothionate and demethylated products. Evidence from algae–bacteria systems supports the view that oxygen enhances complete transformation (Cox 2003; Holmes et al. 2020).

A previous study found that malathion was mineralized to inorganic phosphate in both aerated and non-aerated consortia, although aerated systems generally reached the endpoint sooner (Wang et al. 2023). This indicates that even anoxic waters may support malathion mineralization when appropriate bacterial consortia are present, a finding relevant to treated wetlands and stormwater retention ponds (Wang and Hoffman 1991). Sediment–water interfaces are particularly important because they contain sharp redox transitions and can mediate rapid exchanges between oxic water and more reduced porewater (Singh and Walker 2006). A previous study reported faster malathion loss in seawater–sediment microcosms than in seawater alone in South Carolina coastal samples (Cotham Jr and Bidleman 1989). The difference in malathion degradation was attributed to both sorption onto sediment particles and microbial activity near the sediment surface, as reported in another study (Kulluru et al. 2010). Although several studies have reported that aquatic sediments harbor microbial communities that contribute to malathion attenuation, treating aquatic sediments as the sole sink for attenuation may underestimate malathion biotransformation in the water column (Paris et al. 1975).

### Nutrient availability and co-metabolism

The enzymatic capacity required for malathion degradation can impose a measurable metabolic cost, so the availability of nutrients such as carbon and nitrogen is often an important control on the degradation rate. Nutrient enrichment through natural organic matter inputs, agricultural runoff, wastewater inputs, or experimental amendments can stimulate bacterial growth, increase active biomass, and, in some systems, enhance the expression of degradative enzymes (Buratti et al. 2003; Pillay et al. 2025a). Consistent with this, eutrophic waters and organic-rich sediments often show faster malathion dissipation than oligotrophic systems under otherwise comparable conditions, although the magnitude of this effect depends on community composition and local geochemistry (Bhattacharjee et al. 2026; Thabit and El-Naggar 2013).

Co-metabolism appears to be particularly important for degradation at low malathion concentrations, where the compound may not provide sufficient carbon or energy to support substantial microbial growth (Xie et al. 2013). *Acinetobacter johnsonii* MA19 from aquatic samples in Beijing illustrates this pattern (Shan et al. 2009). The study showed that adding optimized co-metabolic substrates substantially accelerated the aquatic degradation of malathion by MA19, even though the strain did not grow on malathion as a sole carbon source. This result is consistent with organophosphate co-metabolism: when malathion occurs at field-relevant concentrations, often in the µg/L range, complete reliance on it as the sole growth substrate is unlikely, and degradation may depend in part on co-occurring dissolved organic matter. A previous study reported a related pattern for salt-marsh isolates: of 20 strains tested, 11 grew on malathion alone, whereas 4 required peptone amendment to achieve comparable degradation rates (Bourquin 1977).

In oligotrophic systems, including clear lakes, low-dissolved-organic-carbon streams, and some open-ocean settings, microbial activity is often constrained by carbon or nutrient limitation, which can contribute to slower malathion turnover. This may partly explain why persistence in some low-productivity waters can exceed expectations based on laboratory half-lives (Carlson et al. 2007; Del Giorgio and Cole 1998). Continued degradation of malathion and its mono- and dicarboxylic acid derivatives may also release phosphorus into the surrounding environment, which might serve as a phosphorus source in such environments (Stamm et al. 2025a). In contrast, agricultural drainage canals, urban wastewater, and eutrophic ponds typically receive substantial inputs of carbon and nitrogen, support high microbial biomass, and may sustain broader catabolic potential. *Bacillus* sp. FYM31 from agricultural drainage water (Abdelall et al. 2022) and *Acinetobacter baumannii* AFA from domestic sewage (Azmy 2015), which were isolated from nutrient-rich environments, suggest that these strains may have strong degradation potential. Nevertheless, degradation performance depends not only on environmental origin but also on strain-specific traits.

Malathion concentration may also influence biodegradation indirectly by imposing physiological stress on degrading bacteria. In *Shewanella oneidensis* MR-1, it was found that increasing malathion concentrations decreased superoxide dismutase and catalase activities while increasing malondialdehyde content, consistent with elevated oxidative stress and lipid peroxidation (Pan et al. 2024). With prolonged exposure, biomass declined, Na⁺/K⁺-ATPase activity was inhibited, and cells eventually exhibited structural damage and rupture. Accordingly, degradation rates measured in short-term batch assays at high malathion concentrations may not accurately represent the transformation capacity sustainable under chronic contamination, where persistent oxidative stress could reduce the viability and functional performance of degrader populations.

Overall, temperature, pH, redox state, and nutrient availability interactively influence the biotransformation of malathion in the environment. Warm, neutral-to-mildly alkaline, oxic, and organic-rich systems will generally favor rapid malathion degradation, whereas cold, acidic, oxygen-limited, and oligotrophic settings are more likely to support slower transformation and longer persistence. Because most natural environments fall between these extremes, prediction requires consideration of multiple physicochemical controls together, as well as the composition and functional potential of the resident microbial community (Basapuram et al. 2025b).

### Formation, toxicity and fate of malaoxon

Malaoxon is the principal oxidative transformation product of malathion and the metabolite of greatest toxicological concern. It forms by desulfuration of the thiophosphate (P = S) to the corresponding oxon (P = O), catalyzed biologically by microbial monooxygenase and occurring abiotically through photolysis, chemical oxidation, and during water-treatment oxidation (Kralj et al. 2007; Paschal and Neville 1976). Malaoxon exhibits markedly greater acetylcholinesterase inhibition, reported to be 10–40 times more potent than malathion, raising important ecotoxicological implications (Kralj et al. 2007).

The transformation to malaoxon is influenced by various environmental controls. At acidic pH, the metabolite is more persistent than its parent compound, whereas the transformation is 4 times faster at alkaline pH (Health Canada 2023; Kralj et al. 2007; Paschal and Neville 1976; U.S. EPA, 2009). This distinction is relevant for environmental risk assessment in acidic surface waters such as peat-fed streams, soft-water lakes, and some wetlands (Nadalian et al. 2016). Oxygen availability has implications for malaoxon formation (Kralj et al. 2007). Since oxidative desulfuration is mediated by oxygen-dependent enzymes, this route is expected to be reduced under anoxic conditions (Paschal and Neville 1976; Wang et al. 2023).

The United States EPA Reregistration Eligibility Decision (U.S. EPA, 2009) and the Pest Management Regulatory Agency (Health Canada 2023) noted that oxidation during drinking-water treatment can generate malaoxon, which is a direct human-exposure pathway, as treatment can deliver the oxon rather than removing it. Therefore, greater attention to malaoxon as a routinely monitored analyte would improve exposure estimation and risk characterization in malathion–contaminated systems (Dar and Kaushik 2023).

## Ecological implications

Implications for monitoring and managing malathion contamination in aquatic and soil ecosystems have been highlighted in the literature. The consistently emphasized point is that parent-compound monitoring alone cannot capture the toxicological profile of a contaminated system. Hydrolytic transformation produces malathion monocarboxylic acid isomers and malathion dicarboxylic acid as the dominant metabolites (Bourquin 1977; Mali et al. 2022; Paris et al. 1975; Singh et al. 2012a; Sirajuddin et al. 2020; Walker and Stojanovic 1974), and a previous study characterized potassium dimethyl phosphorothioate and potassium dimethyl phosphorodithioate as products of Arthrobacter-mediated degradation in Mississippi sediments (Walker and Stojanovic 1974).

A second implication concerns the source-linkage between wastewater and the receiving aquatic systems. *Bacillus thuringiensis* MOS-5 from Egyptian wastewater achieved approximately 50% degradation over 15 days (Mohamed et al. 2010). *Acinetobacter baumannii* AFA from domestic sewage rapidly degraded malathion in culture (Azmy. 2015), and *Escherichia coli* IES-02 from wastewater achieved 99% removal of 50 mg/L within four hours (Sirajuddin et al. 2020). These findings identify wastewater infrastructure as a source of active malathion-degrading taxa whose performance directly affects effluent quality entering surface waters.

A third implication is that the demonstrated capacity of mixed microbial consortia to mineralize malathion in water systems provides a practical basis for natural attenuation and passive treatment approaches (Bourquin 1977; Cotham Jr and Bidleman 1989; Geed et al. 2016). Algae-bacteria consortia mineralized malathion to inorganic phosphate in both aerated and non-aerated water, an outcome Wang et al. (2023) highlighted as directly relevant to pond and constructed-wetland applications. Finally, recent watershed-scale work in the Oconee River in Georgia demonstrated that anion-mediated abiotic pathways contribute to organophosphate loss alongside biological degradation (Stamm et al. 2025a), implying that ecological risk in riverine systems should incorporate both microbial and nutrient-matrix controls when predicting compound fate and exposure.

### Bioremediation implications

The bacterial capabilities cataloged in Table 3 have important implications for the design of malathion bioremediation strategies, although translating laboratory efficacy into reliable field performance remains slow. The surveyed literature reveals three recurring patterns that collectively provide a robust framework for strain selection, system design, and operational monitoring.

The first concerns community structure, which repeatedly appears to govern degradation performance. A previous study demonstrated complete malathion removal within 15 days using a three-strain consortium of *Bacillus paramycoides*, *Bacillus cereus*, and *Micrococcus aloeverae *(Dar and Kaushik 2023) whereas the same strains in monoculture achieved only 50–68% removal under comparable conditions. Complementary enzyme repertoires among the microbial consortia broaden the range of reactions across the degradation network. Metabolic cross-feeding sustains biomass when the primary substrate becomes limiting, and intermediates that would accumulate are metabolized by other members of the consortium (Gavrilescu 2005; Gupta and Gandhi 2023; Massoud et al. 2007). Accordingly, these findings highlight the use of functionally mixed consortia rather than exclusive dependence on high-performing single strains.

Isolates derived from matrices that resemble the treatment environment generally perform better. *Bacillus* sp. FYM31, isolated from agricultural drainage water, maintained performance in that matrix at concentrations up to 700 mg/L (Abdelall et al. 2022), whereas *Acinetobacter baumannii* AFA, recovered from domestic sewage, degraded malathion efficiently in sewage-derived cultures (Azmy 2015). Although statistical optimization can expand the operating range of a strain, as demonstrated for *Bacillus* sp. AGM5, using Taguchi experimental design (Dar and Kaushik 2022), it cannot overcome the mismatch between strain physiology and site geochemistry (Das and Dash 2014). For this reason, site-specific isolation and screening remain more important than relying on existing culture collections, especially if the target matrix is chemically distinctive. Strain selection should also account for tolerance to malathion-induced oxidative stress, since it was found that even strong degraders such as *Shewanella oneidensis* MR-1 undergo inhibition of antioxidant enzymes, membrane injury, and eventual cell rupture under continuous exposure to high malathion concentrations (Pan et al. 2024).

Treatment plants are cost-effective options for treating malathion-contaminated waters. Algae–bacteria consortia can mineralize malathion under both aerated and non-aerated conditions (Wang et al. 2023), a valuable property for retention ponds, treatment wetlands, and similar systems lacking active aeration. Such systems require minimal investment, energy input, and operating supervision, but demand larger land areas and longer retention times (Das and Dash 2014; Gavrilescu 2005). Treatment plants are therefore better suited for high-volume matrices such as agricultural or surface runoff. In places where more rapid treatment is required, packed-bed and other engineered bioreactor designs provide extended contact time for the strain or consortium to operate. These systems achieve higher and more controllable treatment rates; however, construction, aeration, monitoring, and reinoculation requirements increase operational costs. Packed-bed systems are suitable where contaminant loads are high and volumes are small, such as treated effluents or equipment wash water (Das and Dash 2014; Gupta and Gandhi 2023). Depending on the environment in which the bioremediation strategies are developed, consortia can be tailored to achieve maximum efficacy and efficiency. For example, in cold environments, a tailored consortium, including psychrotolerant bacteria like *Rhodococcus tibetensis* FXJ9.536 (Huang et al. 2022), isolated from high-altitude environments and active at temperatures as low as 10 ℃, can facilitate faster malathion degradation.

Operational considerations or limitations identified across these strategies include monitoring that must extend beyond the parent compound to include transformation metabolites (Health Canada 2023; U.S. EPA, 2009). The accumulation and subsequent degradation depend on the specific microbial community and treatment conditions. Another limitation is that scale-up often reduces process stability. Variations in temperature, pH, dissolved oxygen, nutrient composition, and interaction with indigenous microbiota can each erode the performance of strains optimized in laboratory settings (Wang et al. 2023). A third limitation is economic rather than microbiological: the capital and operating requirements of engineered systems can constrain deployment even where laboratory performance is strong. Cost therefore acts alongside process stability in determining strategies that can be implemented in the field (Gavrilescu 2005). Consequently, pilot-scale validation under field-relevant conditions remains an important step in the development of operational malathion bioremediation systems.

### Future perspectives

Despite the growing number of reported malathion-degrading bacteria, the pathways responsible for transformation and detoxification remain incompletely resolved. Much of the available literature documents parent-compound removal without establishing complete metabolite profiles, mineralization, or residual toxicity. Integrating isotope-tracing experiments and high-resolution mass spectrometry with metagenomic, transcriptomic, and proteomic analyses would help connect active microorganisms and degradation genes with specific transformation products. Such information is particularly important for distinguishing hydrolytic pathways that generally reduce toxicity from oxidative desulfuration pathways that generate malaoxon. Concurrent analysis of malathion, malaoxon, other transformation products, and residual toxicity would therefore provide a more reliable assessment of biodegradation outcomes than parent-compound disappearance alone. Important knowledge gaps also remain regarding the organization and regulation of degradation genes, their association with mobile genetic elements, and the structural basis of enzyme specificity. Protein engineering, immobilized biocatalysts, and defined microbial consortia represent promising approaches for improving catalytic stability and downstream metabolite transformation, although their performance in complex environmental matrices remains poorly characterized. Progress toward practical application will depend on validation in natural water and sediment systems under realistic variations in pH, temperature, redox conditions, nutrients, co-contaminants, and pesticide loading. Mesocosm and pilot-scale studies that incorporate transformation-product monitoring, mineralization, toxicity reduction, operational stability, and cost would clarify when biological treatment provides a scalable advantage over natural attenuation or abiotic treatment technologies.

## Conclusion

Bacterial biotransformation is a major control on malathion persistence in many aquatic and sedimentary environments. Its relative importance also depends on matrix-specific abiotic and biological conditions. The predominant microbial transformation pathway reported in bacteria is carboxylesterase-mediated hydrolysis, which converts malathion to mono- and dicarboxylic acid derivatives that may undergo further biodegradation and more extensive mineralization. A parallel oxidative pathway, mediated by oxygen-dependent enzymes, can generate malaoxon, a metabolite generally considered more toxic than the parent compound, whose environmental fate is strongly influenced by pH and redox conditions. Diverse bacterial taxa have been reported to participate in these transformations, including members of *Bacillus*, *Pseudomonas*, *Acinetobacter*, *Micrococcus*, *Rhodococcus*, *Sphingomonas*, *Arthrobacter*, *Brevibacillus*, *Lysinibacillus*, *Ochrobactrum*, *Pseudidiomarina*, and *Escherichia*. Reported degradation performance varies widely, and this variation appears to reflect substrate concentration, strain-specific physiology, experimental design, and environmental matrix.

Further research into integrating community-level data from gene sequencing and metagenomics with functional measurements, such as enzyme assays and isotope-labeled substrate experiments, would help connect the extensive strain-level literature needed to understand exposure and risk. Additionally, greater attention to malaoxon as a monitored analyte would improve risk assessment, as its persistence may depend on the matrix’s redox conditions. The scale-up of consortium-based bioremediation through site-specific isolation, process optimization, and pilot-scale validation remains an understudied yet potentially practical strategy for managing chronic malathion contamination, especially in regions where treatment infrastructure can be developed. Progress in these areas would substantially improve the ability to predict, manage, and remediate malathion contamination across diverse environmental matrices.

## Data Availability

No datasets were generated or analysed during the current study.
